# Genetic and phenotypic characterizations of IncX3 plasmids harboring *bla*_NDM-5_ and *bla*_NDM-16b_ in Japan

**DOI:** 10.1128/spectrum.02167-23

**Published:** 2023-10-19

**Authors:** Hui Zuo, Yo Sugawara, Shizuo Kayama, Sayoko Kawakami, Koji Yahara, Motoyuki Sugai

**Affiliations:** 1 Antimicrobial Resistance Research Center, National Institute of Infectious Diseases, Tokyo, Japan; University of Pretoria, Pretoria, South Africa

**Keywords:** *bla*_NDM_, IncX3, JARBS

## Abstract

The spread of the gene encoding the NDM-type carbapenemase, *bla*_NDM_, poses a serious threat to clinical practice and public health. This gene is usually carried by transferable plasmids, which facilitates its rapid spread among various species of Enterobacterales. Plasmids with the IncX3 replicon play a major role in the spread of *bla*_NDM_ in Asian countries. In this study, we conducted genomic sequencing and a comparative analysis of the IncX3-type plasmids harboring *bla*_NDM-5_ and *bla*_NDM-16b_, a single-nucleotide variant of *bla*_NDM-5_, isolated in a national antimicrobial-resistant bacterial genomic surveillance in Japan (Japan Antimicrobial Resistant Bacterial Surveillance), which was carried out during 2019–2020. We compared five plasmids harboring *bla*_NDM-5_ and one plasmid harboring *bla*_NDM-16b_. They were highly identical and differed in several nucleotide substitutions or variations in insertion sequences, although they were carried by different species or genotypes. Notably, *bla*_NDM-16b_-carrying pJBBDACG-19-0070 was identical to a plasmid carrying *bla*_NDM-5_ except for one nucleotide substitution in the *bla*_NDM_ gene throughout the 46 kb sequences. Transformants carrying these two plasmids showed comparative resistance to carbapenems, indicating that a substitution on the NDM enzyme (Ala233 to Val) did not affect carbapenem resistance, at least under normal experimental conditions. A database search revealed that several identical plasmids carrying *bla*_NDM-5_ were reported from neighboring countries, mainly China, before our surveillance period. Our analysis suggested that the IncX3 plasmid harboring *bla*_NDM-5_ is spreading in Japan, and *bla*_NDM-16b_ emerged from a single nucleotide substitution on the plasmid, with widespread dissemination in Asian countries.

**IMPORTANCE:**

IncX3 plasmids harboring *bla*_NDM-5_ play a major role in the spread of carbapenem resistance in Asia, particularly in China, in clinical and environmental settings. In this study, we present that Enterobacterales isolates carrying IncX3 plasmids harboring *bla*_NDM-5_ have been disseminated in Japan, where their identification was previously rare. In addition, *bla*_NDM-16b_, a single-nucleotide variant of *bla*_NDM-5_, was found to be carried by an identical IncX3 plasmid. A comparative sequence analysis revealed that the *bla*_NDM-16b_ gene emerged from a single nucleotide substitution on an IncX3 plasmid harboring *bla*_NDM-5_. The *bla*_NDM-16b_ gene did not confer elevated carbapenem resistance compared to *bla*_NDM-5_ in our assay using transformants carrying the plasmid harboring either of these genes, although the A233V substitution was reported to confer stability to the enzyme in ion-depleted conditions. Nevertheless, vigilance regarding the emergence of novel variants is required.

## OBSERVATION

New Delhi metallo-β-lactamase (NDM) confers resistance to all β-lactams except monobactams, which has been met with significant public health concern because the gene encoding NDM (*bla*_NDM_) is usually carried by transferable plasmids and often coexists with many other resistance determinants (1). *bla*_NDM_ genes are rapidly spreading, particularly in Asian countries, and have been detected in various sources outside clinical settings, such as sewage, livestock, and even food samples from South, East, and Southeast Asian countries (2 – 5), indicating their presence in the community. One of the major vehicles for the gene is the IncX3-type plasmid, which is highly conjugative and broadly disseminated, particularly in China. In Japan, identification of the *bla*_NDM_ gene is still rare, and *bla*_NDM-1_ and *bla*_NDM-5_ have been sporadically reported in clinical isolates since their first isolation (6, 7). In this study, we report the isolation and comparative analysis of IncX3-type plasmids harboring *bla*_NDM-5_ and *bla*_NDM-16b_, a single nucleotide variant of *bla*_NDM-5_, in a Japan Antimicrobial Resistant Bacterial Surveillance (JARBS) conducted during 2019–2020 (8).

**FIG 1:**
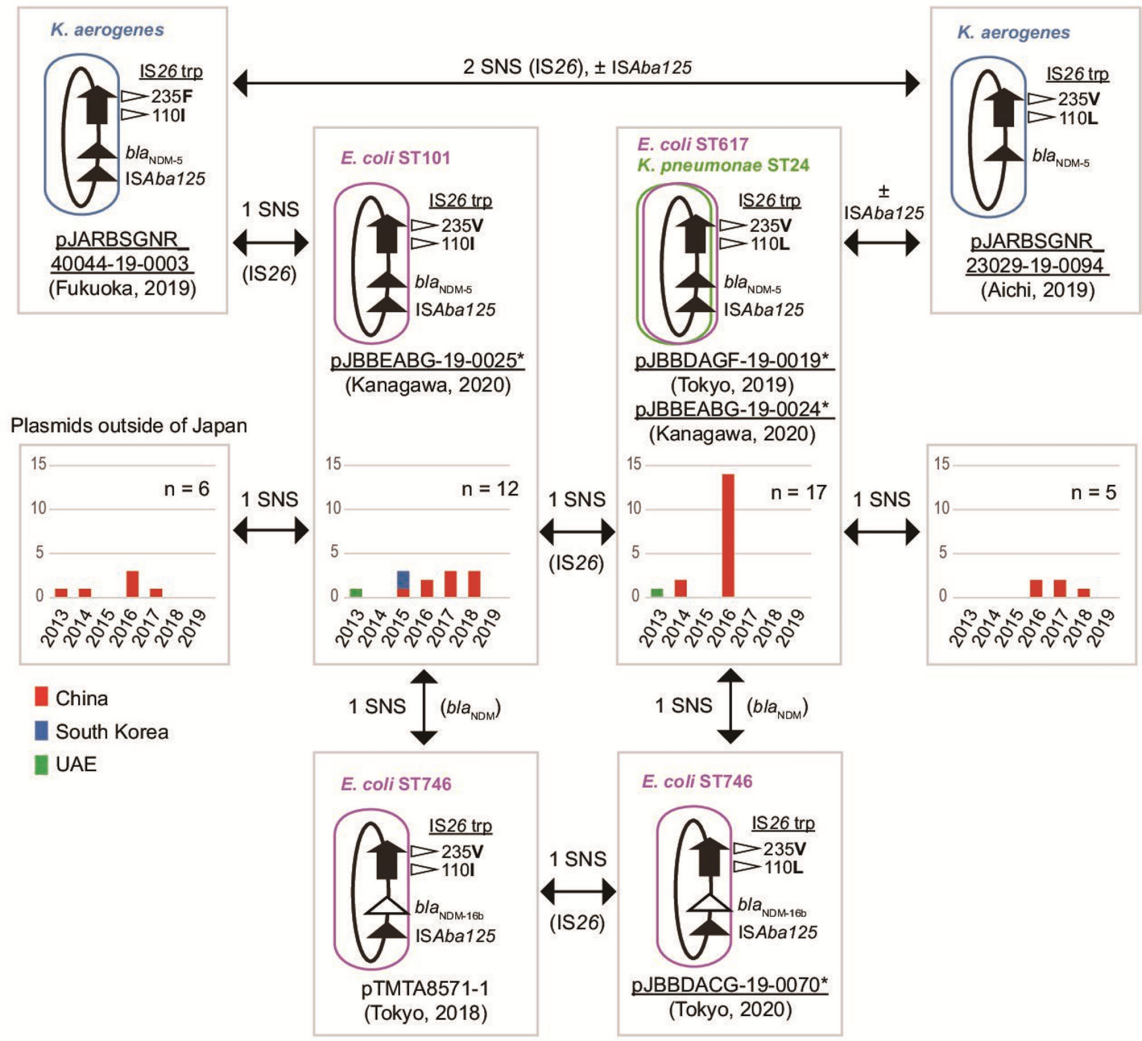
Relationship between the IncX3 plasmids carrying *bla*_NDM-5_ or *bla*_NDM-16b_. Plasmids obtained during our surveillance are underlined, and those marked with an asterisk were obtained in this study. SNS denotes single nucleotide substitutions on IS*26*, *bla*_NDM_, or at any position of the plasmid if there are no specifications. The 110th and 235th amino acid residues of IS*26* transposase (trp), *bla*_NDM_ genes, and the presence of IS*Aba125* insertions are shown in the figure. Graphs show the number and year of isolation of identical plasmids deposited in GenBank from other countries. Red, China; blue, South Korea; green, the United Arab Emirates (UAE).

In a nationwide surveillance focusing on Gram-negative bacteria (JARBS-GNR), clinical Enterobacterales isolates with reduced carbapenem susceptibility were collected from 175 hospitals (https://www.niid.go.jp/niid/ja/from-amrc.html). Isolates positive for known carbapenemase genes were subjected to Illumina and Oxford Nanopore sequencing, followed by hybrid assembly using Unicycler 0.4.8 to obtain their complete sequence. The detection of antimicrobial resistance determinants and multilocus sequence typing was performed using an in-house bioinformatics pipeline. Among these isolates, we focused on those carrying the IncX3-type plasmid harboring *bla*_NDM_: five isolates carrying *bla*_NDM-5_, including two previously reported isolates (9), and one carrying *bla*_NDM-16b_. The *bla*_NDM-16b_ gene differs from *bla*_NDM-5_ by a single nucleotide substitution, which results in the substitution of alanine to valine at the 233rd amino acid residue (Fig. 1). The isolate carrying *bla*_NDM-16b_, designated JBBDACG-19-0070, was an *Escherichia coli* sequence type (ST) 746, which was isolated from a middle-stream urine specimen. The isolate contained several antimicrobial resistance genes, conferring resistance to a β-lactam (*bla*_CTX-M-14_), aminoglycosides [*aph(3″)-Ib*, *aph (6)-Id*, and *aadA5*], macrolide (*mphA*), trimethoprim (*dfrA17*), sulfanilamides (*sul1* and *sul2*), fluoroquinolones (*mdfA*), phenicol (*floR*), tetracycline [*tet(A*)], and *bla*_NDM-16b_. All of these apparently underlie the antimicrobial resistance phenotype (Table 1). These characteristics of JBBDACG-19-0070 were almost identical to those of the first *bla*_NDM-16b_-positive isolate in Japan, TA8571 (10). In addition, the plasmid carrying *bla*_NDM-16b_, pJBBDACG-19-0070 (46, 161 bp), was identical to the TA8571 plasmid, pTMTA8571-1, except for one nucleotide substitution in the IS*26* transposase coding region which results in an amino acid substitution (I110L) (Fig. 1).

**TABLE 1:** Antimicrobial susceptibility patterns of NDM-producing isolates and transformants

Antimicrobial agents	MICs (mg/L)				
	JBBDACG-19-0070 (*bla*_NDM-16b_)	pJBBDAGF-19-0019 (*bla*_NDM-5_)	HST08:: pJBBDACG-19-0070 _NDM-16b	HST08:: pJBBDAGF-19-0019 _NDM-5	HST08
Meropenem^*a*^	128	64	32	32	≦0.125
Imipenem^*a*^	128	64	64	32	≦0.125
Doripenem	>8	>8	>8	>8	≦0.5
Piperacillin	>64	>64	>64	>64	≦4
Ampicillin/sulbactam	>32/16	>32/16	>32/16	>32/16	≦4/2
Tazobactam/piperacillin	>64	>64	>64	>64	≦4
Aztreonam	16	2	≦1	≦1	≦1
Ceftazidime	>16	>16	>16	>16	≦1
Cefepime	>16	>16	>16	>16	≦1
Cefoperazone/sulbactam	>32/16	>32/16	>32/16	>32/16	≦8/4
Cefozopran	>16	>16	>16	>16	≦1
Tobramycin	2	>8	<=1	≦1	≦1
Amikacin	8	>32	≦4	≦4	≦4
Gentamicin	2	>8	≦1	≦1	≦1
Fosfomycin	≦4	>16	≦4	≦4	≦4
Colistin	≦1	≦1	≦1	≦1	≦1
Minocycline	4	8	≦1	≦1	≦1
Trimethoprim/sulfamethoxazole	>2/38	>2/38	≦1/19	≦1/19	≦1/19
Ciprofloxacin	>4	>4	≦0.25	≦0.25	≦0.25
Levofloxacin	>8	>8	≦0.5	≦0.5	≦0.5
Chloramphenicol	>16	>16	≦8	≦8	≦8
Tigecycline	≦0.25	≦0.25	≦0.25	≦0.25	≦0.25

^*a*^ The minimum inhibitory concentration (MIC) was determined using the broth microdilution method.

Comparative sequence analysis of these IncX3 plasmids, six from our surveillance and pTMTA8571-1, showed that they were highly identical and differed by minimum single nucleotide substitutions, even though they originated from different hospitals and were identified in various STs or species. Differences among them were classified into four categories: substitutions at either of the two sites on the IS*26* transposase coding region, the presence or absence of IS*Aba125*, and a substitution on the *bla*_NDM_ gene. The two *bla*_NDM-5_-carrying plasmids, pJBBDAGF-19-0019 and pJBBEABG-19-0024, were identical, and the pJBBEABG-19-0025 plasmid differed from these two plasmids by a single nucleotide on the IS*26* coding sequence. pJBBEABG-19-0025 differed from another *bla*_NDM-5_-carrying plasmid from *Klebsiella aerogenes*, pJARBSGNR_440044-19-0003, by another nucleotide in the IS*26* sequence. pJARBS-GNR_23029-19-0094 lacked IS*Aba125* compared to pJARBSGNR_440044-19-0003, as previously reported (9). Notably, *bla*_NDM_-_16b_-carrying plasmids were identical to one of those carrying *bla*_NDM-5_ except for the *bla*_NDM_ gene. pJBBDACG-19-0070 and pTMTA8571-1 were completely identical to plasmids pJBBDAGF-19-0019 and pJBBEABG-19-0025, respectively, except for a substitution on *bla*_NDM_ that yielded an NDM A233V substitution. Identical plasmids were also identified in the public databases. We searched for plasmid sequences similar to our plasmids using NCBI BLAST and included 40 sequences in our analysis. We found 17 and 12 sequences completely identical to pJBBDAGF-19-0019 and pJBBEABG-19-0025, respectively, which were isolated from China, South Korea, and the United Arab Emirates before our surveillance period (Fig. 1; Table S1). Several plasmid sequences with single nucleotide substitutions compared to these plasmids were also deposited, mainly from China, indicating that these identical plasmids were disseminated in these Asian countries.

As shown above, the *bla*_NDM-16b_-carrying plasmid pJBBDACG-19-0070 and the *bla*_NDM-5_-carrying plasmid pJBBDAGF-19-0019 were identical except for a single nucleotide substitution on *bla*_NDM_, which allowed us to conduct a comparative analysis between *bla*_NDM-5_ and *bla*_NDM-16b_ on the same IncX3 plasmid background. Plasmid DNA was extracted from isolates carrying these two plasmids and introduced into the *E. coli* strain HST08 using electroporation. The size and location of the *bla*_NDM_ gene in the transformants were confirmed by S1-nuclease pulsed-field gel electrophoresis, followed by Southern hybridization with a digoxigenin-labeled (Roche, Burgess Hill, United Kingdom) *bla*_NDM_-specific probe (11). The minimum inhibitory concentrations (MICs) of the transformants and their parental strains were determined using broth microdilution for meropenem and imipenem and by MicroScan WalkAway for the other β-lactams. The results were comparable between the transformants, and both the IncX3-*bla*_NDM-5_ and IncX3-*bla*_NDM-16b_ plasmids conferred the same level of resistance to the β-lactams tested on the susceptible parental strain.

In this study, we showed that highly similar IncX3 plasmids harboring *bla*_NDM-5_ were disseminated in Japan and its neighboring countries. Amino acid substitutions at 110th and 235th on IS*26* transposase were the major difference among these plasmids. It is unclear whether these substitutions have occurred once each or several times independently during the spread of these plasmids. The 110th amino acid is located in the catalytic domain of the transposase, and leucine is well conserved at this position in IS*6*/IS*26* family transposases (12). The effect and significance of substitution at this residue by isoleucine are unclear, although the substitution apparently does not affect the hydrophobic characteristics at this position. Reports of IncX3 plasmids with *bla*_NDM-16b_ are still rare, even though their sequence is identical to those carrying *bla*_NDM-5_, suggesting that *bla*_NDM-16b_ recently emerged from *bla*_NDM-5_ on the IncX3 plasmid. Our MIC assay showed that NDM-16b and NDM-5 confer comparable resistance against meropenem on the host bacterium. The result is consistent with a previous report in which the assay was performed using bacteria harboring the *bla*_NDM_ genes cloned into a commercially available vector (13). Meanwhile, an alanine-233 to valine substitution on NDM-5 is known to confer stability to the enzyme under zinc-limited conditions (13, 14). Therefore, it could be beneficial for bacteria to produce NDM-16b rather than NDM-5 to survive iron-depleted conditions, such as at infection sites. Given that this is the case, an organism probably obtained a single nucleotide substitution causing the alanine-233 to valine mutation, that is, *bla*_NDM-5_ to *bla*_NDM-16b_ evolution, but not vice versa, during its spread in clinical settings or under antimicrobial exposure. Notably, *bla*_NDM-16b_ was found in a relatively highly multidrug-resistant strain, *E. coli* ST746. Collectively, our analysis supported the notion that *bla*_NDM-16b_ emerged from IncX3 plasmids harboring *bla*_NDM-5_, which was recently suggested by another research group (10).

In summary, we showed that highly identical IncX3 plasmids harboring *bla*_NDM-5_ are spreading throughout Japan. These plasmids were disseminated in neighboring countries, mainly China, suggesting that Japanese IncX3 plasmids originated in these countries. Although sequence variation among these plasmids was highly limited, one of them occurred in the *bla*_NDM-5_ gene, yielding a new variant, *bla*_NDM-16b_. This substitution did not confer elevated resistance to the host bacterium, as we showed in this study; however, such a substitution might occur at other nucleic acid positions and increase the enzymatic activity of the encoded NDM. Thus, the emergence of novel variants is conceivable, and continued vigilance should be exercised to prevent their further spread.

## Data Availability

The following are available from the DDBJ/ENA/GenBank under the BioProject accession number PRJDB10842: DRR387217/DRR393658 for the *bla*_NDM-16b_ isolate JBBDACG-19-0070 and DRR387527/DRR393677, DRR387702/DRR393709, and DRR387703/DRR393710 for the *bla*_NDM-5_ isolate JBBDAGF-19-0019, JBBEABG-19-0024, and JBBEABG-19-0025, respectively.

## SUPPLEMENTAL MATERIAL

Table S1 spectrum.02167-23-s0001.xlsx Publicly available plasmids analyzed in this study.

ASM does not own the copyrights to Supplemental Material that may be linked to, or accessed through, an article. The authors have granted ASM a non-exclusive, world-wide license to publish the Supplemental Material files. Please contact the corresponding author directly for reuse.
